# Diet Matters: Endotoxin in the Diet Impacts the Level of Allergic Sensitization in Germ-Free Mice

**DOI:** 10.1371/journal.pone.0167786

**Published:** 2017-01-04

**Authors:** Martin Schwarzer, Dagmar Srutkova, Petra Hermanova, Francois Leulier, Hana Kozakova, Irma Schabussova

**Affiliations:** 1 Laboratory of Gnotobiology, Institute of Microbiology of the Czech Academy of Sciences, v. v. i., Novy Hradek, Czech Republic; 2 Institut de Génomique Fonctionnelle de Lyon (IGFL), Ecole Normale Supérieure de Lyon, CNRS UMR 5242, Iniversité Claude Bernard Lyon 1, Lyon, France; 3 Institute of Specific Prophylaxis and Tropical Medicine, Medical University of Vienna, Vienna, Austria; Virginia Polytechnic Institute and State University, UNITED STATES

## Abstract

Germ-free animals have been used to define the vital role of commensal bacteria on the maturation of the host immune system. However, the role of bacterial residues in diet in this setting is poorly understood. Here we investigated the effect of bacterial contamination in sterile diet on the level of allergic sensitization in germ-free mice. Sterile grain-based diets ST1 and R03 were tested for the level of bacterial contamination. ST1 contained higher amount of bacterial DNA, approximately ten times more endotoxin, and induced higher, TLR4-dependent, cytokine production in dendritic cells compared to R03. In a germ-free mouse model of sensitization to the major birch pollen allergen Bet v 1, feeding on ST1 for at least two generations was associated with decreased production of allergen-specific IgE and IgG1 antibodies in sera in comparison to R03. Furthermore, reduced levels of allergen-specific and ConA-induced cytokines IL-4, IL-5 and IL-13 accompanied by increased levels of IFN-γ were detected in splenocytes cultures of these mice. Our results show that contamination of experimental diet with bacterial residues, such as endotoxin, significantly affects the development of allergic sensitization in germ-free mice. Therefore, careful selection of sterile food is critical for the outcomes of germ-free or gnotobiotic experimental models of immune-deviated diseases.

## Introduction

Reduced exposure to exogenous stimuli and/or altered composition of intestinal microbiota due to the overuse of antibiotics, western diet, and reduced prevalence of infection diseases during childhood are feasible factors of increasing prevalence of allergic disorders [1–3]. This concept was first put forth by the hygiene hypothesis and suggested a causal link between allergy and western lifestyle, where the limited exposure to microbes can lead to compromised regulation of the immune responses [4].

In this context, exposure to microbes or microbial components have been associated with protection against allergy later in life [5–7]. One example of such microbe-derived environmental factor is lipopolysaccharide (LPS), ubiquitously present cell wall component of Gram-negative bacteria. LPS and its bioactive moiety endotoxin have been used as a surrogate of microbial burden in the environment [8]. Although the levels of human exposure to LPS are highly variable, they are unavoidable. Several clinical studies have shown that continuous exposure of humans to LPS has protective effects against the development of allergy [5,8–10]. Similarly, LPS prevented an allergic outcome in several experimental models [11–13]. Along these lines, LPS of *Acinetobacter lwoffii*, a Gram-negative bacteria isolated from the farm cowshed, was identified as a protective factor against allergy [14,15].

LPS is a strong immunogen that triggers the activation of innate and acquired immunity via the transmembrane TLR4-mediated signaling [16]. Stimulation of antigen presenting cells, such as dendritic cells (DC) with LPS leads to their maturation associated with increased expression of costimulatory molecules and production of cytokines [17]. In addition, LPS-exposed dendritic cells stimulate the generation of Th1 immune responses associated with production of proinflammatory cytokines, such as IFN-γ. The property of LPS to redirect immune responses from a Th2 towards Th1 immunity has been suggested as a key mechanism of the LPS-induced beneficial effects, influencing the development and maintenance of allergic diseases [18]. Bortolatto *et al*. have demonstrated that LPS impairs the development of allergic Th2 responses via the IL-12/IFN-γ axis and this effect was TLR4-dependent [19]. Similarly, Rodriguez *et al*. reported that LPS reduced allergic Th2 responses in mice via the TLR4-dependent pathway [11].

Germ-free (GF) mice that lack any exposure to living pathogenic or nonpathogenic microorganisms, provide an attractive model to investigate the role of the composition and function of intestinal microbiota on the development of food allergy, allergic airway inflammation, or allergen-specific tolerance induction [20]. It has been shown that GF mice are more responsive to allergic sensitization, exhibit dysregulated allergic airway inflammation, and display higher levels of serum allergen-specific IgG1 and IgE with increased production of Th2-associated cytokines compared to the animals colonized by microbiota [17,20–25]. Data from our lab have shown that this exacerbated allergic sensitization in GF mice can be prevented by mother-to-offspring colonization of GF mice with single probiotic strain *B*. *longum* [17] or by colonization of adult GF mice with a mixture of three *Lactobacillus* strains [26]. Although the gastrointestinal tract of GF animals can be considered sterile, it is still permanently exposed to self-antigens, ingested food antigens [27] and microbial residues in sterile food or beddings, such as endotoxin. As far as the bacterial contamination in food is concerned, bacterial residues in sterile chow of GF mice have been associated with expansion of B and T cells in the gut associated lymphoid tissue (GALT) and with higher levels of Th1 cytokine IL-12 and lower levels of Th2 cytokine IL-4 upon mitogen stimulation of spleen cells in comparison to control mice on LPS-free diet [28]. This data suggest that the contamination of sterile food with bacterial residues may influence the outcome of experimental models of Th1/Th2-associated diseases performed on GF animals. However, this premise has not been explored to date.

Here, we expand on our previous observations and show that not only the colonization of GF animals with commensal bacteria but also exposure to bacterial residues (endotoxin) present in sterile food is able to modulate the functional maturation of immune system leading to altered responses in an experimental model of allergic sensitization. Furthermore, this is the first demonstration of specific effects of different diets on the sensitization in germ-free mice.

## Materials and Methods

### Mouse diets, diet extract preparation and measurement of LPS contamination

ST1 (Velaz, Praha, Czech Republic) and R03 (SAFE, Augy, France) are both grain based diets which have been routinely used after irradiation to feed GF animals [17,24]. Composition of the R03 diet can be found on vendor’s web page www.safe-diets.com, composition of the ST1 diet is in supplementary material (S1 Table). Both diets are nutritionally adequate and animal growth curves are comparable. For the preparation of extracts (eST1 and eR03), sterile pellets were grounded by LPS-free sterile scissors, extensively vortexed and sonicated on ice for 5 minutes. Supernatants were collected after centrifugation, filtered (0.2 μm) and LPS concentrations were determined by the fluorescent PyroGene™Recombinant Factor C Assay (Lonza, Switzerland) according to the manufacturer’s instructions.

### DNA isolation and 16S rDNA PCR amplification

Sterile diet (200 mg) was homogenized by the Tissue lyser (Quiagen, Hilden, Germany) in 0.6 ml Tris-EDTA buffer (10 mM Tris-HCl, 5 mM EDTA, pH 7.8) for 10 min/50 Hz and centrifuged (200 g/5 min). Supernatant (200 μl) was washed with 400 μl of Tris-EDTA buffer and centrifuged again at 200 g/5 min. Supernatant (350 μl) was resuspended in 500 μl of lysis buffer (10 mM Tris–HCl, 5 mM EDTA, pH 7.8) containing lysozyme (6 mg/ml). After 1 h incubation at room temperature, 25 μl of 20% SDS and 10 μl of proteinase K (100 μg/ml) was added to each sample and incubated at 55°C overnight. Finally, mixture was treated with 10 μl RNase A (10 μg/μl) for 30 min at 37°C. DNA was isolated by phenol-chloroform extraction and dissolved in 50 μl Tris-EDTA buffer. The purity, integrity and concentration of nucleic acids were confirmed by agarose gel electrophoresis and UV spectrophotometry as previously described [29]. Bacterial 16S rDNA was amplified using PCR with the universal primers 27F (5′ AGA GTT TGA TCC TGG CTC AG 3′) and 1492R (5′ GGT TAC CTT GTT ACG ACT T 3′) as previously described [21]. To exclude false negative results caused by inhibitors in the sample, 10x and 100x dilutions of the original sample were used as a template. Ten ng of chromosomal DNA from *Escherichia coli* was used as a positive control. Amplification products were separated by 1.2% agarose gel electrophoresis, visualized using GelRed^TM^ Nucleic Acid Gel Stain (Biotinum, Hayward, CA, USA) and images were obtained by Fluorescent Image Analyser FLA-7000 (Fujifilm Corporation, Tokyo, Japan).

### Animals

Germ-free BALB/c mice were kept under sterile conditions in Trexler-type plastic isolators and supplied with water and sterile pellet diet ST1 or R03 *ad libitum*. Both diets were sterilized by irradiation. Fecal samples were weekly controlled for microbial contamination as previously described [30]. TLR4-/- deficient mice on BALB/c background [31] were a kind gift from M. Freudenberg (Freiburg, Germany). BALB/c and TLR4-/- deficient mice were kept under specific pathogen-free (SPF) conditions and fed sterile pellet ST1 diet. The animal experiments were approved by the Committee for the Protection and Use of Experimental Animals of the Institute of Microbiology v.v.i., Academy of Sciences of the Czech Republic (approval ID: 50/2013).

### Experimental design

Germ-free mice were kept on the respective diets for at least two generation. Eight-week-old GF female mice were subcutaneously (s.c.) sensitized on days 1, 14 and 28 with 1 μg of Bet v 1 (Biomay, Vienna, Austria) emulsified in 100 μl of Al(OH)_3_ (Serva, Germany). Mice sham treated with Al(OH)_3_ alone were used as controls. Seven days after the last immunization, mice were killed by CO_2_ asphyxia and samples were taken for further analysis.

### Humoral immune responses

Blood samples were taken at sacrifice and serum levels of anti-Bet v 1 IgE, IgG1, IgG2a and IgA were measured by ELISA as previously described [32]. The measurement of the results for each Ig subtype has been performed on the same plate and results were reported as optic density (OD). The activity of Bet v 1-specific IgE in serum was measured by rat basophile leukemia cells degranulation assay as described previously [33]. Levels of total IgE and IgA in serum were measured by a commercial ELISA kit as recommended by the manufacturer (Bethyl, USA). Small intestine was excised and faeces removed by flushing the lumen with 2 ml of cold PBS. The intestine was cut open lengthwise and frozen in 1 ml of Complete protease inhibitor in PBS (Roche, Manheim, Germany). After thawing, samples were incubated in 20% saponine solution (Sigma-Aldrich) overnight to permeabilize cell membranes. Supernatants were collected after centrifugation (2000 g; 10 min) and stored at –20°C. Levels of Bet v 1 specific IgA and IgG1 in gut lavage were measured as described above and reported as OD.

### Cellular immune responses

Spleen single cell suspensions from sensitized and control mice on ST1 or R03 diets were prepared and cultured as previously described [33]. Mononuclear cells (3 x 10^6^ cells/ml) were stimulated with Bet v 1 (20 μg/ml), ConA (1.5 μg/ml; Sigma-Aldrich, USA) or media alone in 96-well plates at 37°C for 60 hours in culture medium (RPMI 1640 supplemented with 10% heat-inactivated FCS, 2 mM L-glutamine, 100 U/ml penicillin, 100 μg/ml streptomycin). Levels of cytokines in culture supernatants were measured by the MILLIPLEX MAP Mouse Cytokine/Chemokine Panel (Millipore, USA) according to manufacturer’s instructions and analyzed with the Bio-Plex System (Bio-Rad Laboratories, USA). Values are expressed as pg/ml or ng/ml after subtraction of baseline levels of unstimulated cultures.

### Preparation and activation of bone marrow-derived dendritic cells

Mouse bone marrow-derived DC (BM-DC) from wild-type BALB/c and TLR4-/- mice were prepared as previously described [34]. Briefly, the bone marrow precursors were isolated from femur and tibia of respective mice. Cells were cultured at 4x10^5^/ml in bacteriological Petri dishes in 10 ml culture medium with GM-CSF (20 ng/ml; Sigma-Aldrich). Fresh medium was added at day 3 and 6 and BM-DC were used on day 8 of culture. BM-DC (10^6^ cells/well) were stimulated with 100 μg/ml of ST1 or R03 diet extracts for 18 h. As controls, BM-DC were incubated with ultrapure LPS (LPS-EB, 1 μg/ml, InvivoGen, USA). Levels of IL-10, IL-12p70, TNF-α and IL-6 in culture supernatants were determined by ELISA Ready-Set-Go! kits (eBioscience, USA) according to manufacturer’s instructions.

### Stimulation of HEK293 cells stably transfected with TLR

HEK293 cells stably transfected with plasmid carrying human (h)TLR2/CD14 gene were kindly provided by M. Yazdanbakhsh (Leiden, Netherlands) and cells transfected with hTLR4/MD2/CD14 were a gift of B. Bohle (Vienna, Austria). Cells were stimulated with ST1 or R03 diet extracts (10 or 100 μg/ml). TLR2 ligand Pam3Cys (Pam3CSK4, 1 μg/ml, InvivoGen, USA) and TLR4 ligand LPS (LPS-EB, 1 μg/ml, InvivoGen, USA) were used as positive controls. After the 20-h incubation period, culture supernatants were harvested and concentration of human IL-8 was analyzed by ELISA (Thermo Scientific, USA) according to the manufacturer’s instructions.

### Statistical analysis

Data are expressed as means ± SEM. Statistical analysis was performed by non-parametric Mann–Whitney U-test using GraphPad Software (GraphPad Prism 5.04, San Diego, USA); P values < 0.05 were considered significant.

## Results

### Bacterial DNA contamination and endotoxin content differs considerably between two sterile diets commonly used in germ-free animal facilities

**Fig 1:**
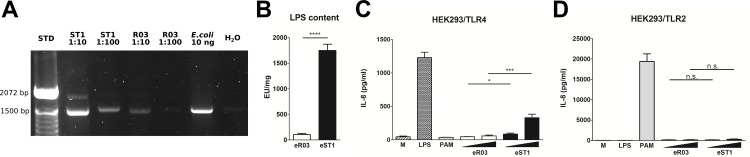
Bacterial contamination differs between R03 and ST1 diets. (A) DNA isolated from R03 and ST1 diets was diluted in ddH_2_O (1:10 and 1:100) and PCR was performed with Bacteria-specific primers. PCR products were separated by 1.2% agarose gel electrophoresis. *E*. *coli* DNA was used as positive control, H_2_O as negative control. (B) Endotoxin levels in R03 and ST1 diet extracts (eR03, eST1 respectively) were determined by the fluorescent PyroGene™Recombinant Factor C Assay. Data are plotted as mean values ± SEM. Representative results from five independent experiments are shown. (C-D) Human embryonic kidney cells (HEK293) stably transfected with an expression vector for human TLR4 (HEK293-hTLR4/MD2/CD14; HEK293/TLR4) and TLR2 (HEK293-hTLR2/CD14; HEK293/TLR2) were cultured for 20 h with 10 μg/ml or 100 μg/ml of respective diet extracts (eR03, eST1). Ultra-pure lipopolysaccharide from *E*. *coli* (LPS; 1 μg/ml) and Pam3CSK4 (PAM, 1 μg/ml) were used as positive controls for TLR4 and TLR2, respectively. Unstimulated cells (M) were used as controls. Results are expressed as mean values ± SEM, representative results from three independent experiments are shown. *P ≤ 0.05, **P ≤ 0.01, ***P ≤ 0.001, n.s = not significant.

Bacterial residues in sterile diets may influence maturation of immune system in GF mice [28]. We therefore tested the presence of bacterial material in two different diets which are routinely used in gnotobiotic breeding facilities. First, the presence of bacterial DNA in the sterile diets R03 and ST1 was investigated by PCR. The data show that only low levels of bacterial DNA are present in the R03 diet (Fig 1A). On the contrary, ST1 diet contains higher levels of bacterial DNA, detected in sample diluted 1:10 or even at higher dilution 1:100 (Fig 1A). Second, the amount of endotoxin was measured in extracts of both diets (eR03 and eST1) by the PyroGene™Recombinant Factor C Assay. The data clearly indicate that the level of endotoxin contamination differs significantly between the two diets, with the levels detected in eR03 being 10 times lower compared to the levels of endotoxin in eST1 (Fig 1B). Along these lines, the presence of TLR4 or TLR2 ligands in both diets was tested by HEK293 cells transfected with respective receptor. No significant production of IL-8 was detected in the HEK293/TLR4 or HEK293/TLR2 cultures incubated with eR03, suggesting the lack of TLR4 or TLR2 ligands in this diet (Fig 1C and 1D). On the other hand, eST1 induced dose dependent production of IL-8 by HEK293/TLR4 indicating the contamination with TLR4 ligand (Fig 1C and 1D).

### TLR4 ligands are the main component in the diet extracts driving the cytokine production and maturation of dendritic cells

**Fig 2:**
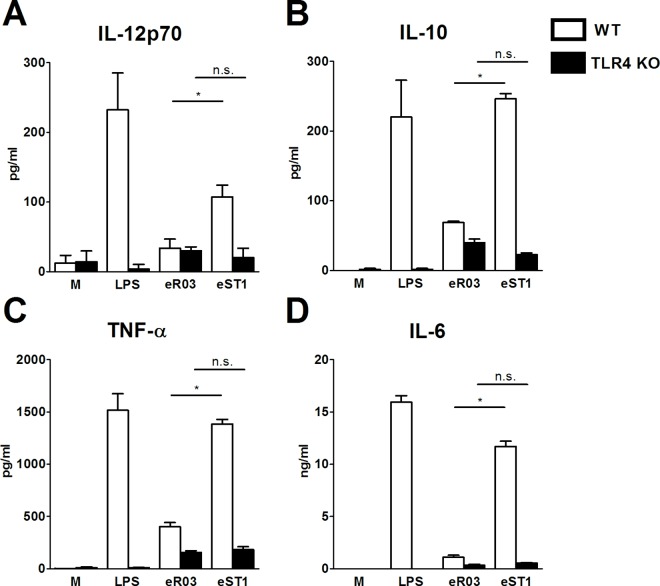
Extracts derived from R03 and ST1 diets induce different cytokine production from dendritic cells. Bone marrow-derived dendritic cells (BM-DC) generated from wild-type (WT, white bars) and TLR4-deficient (TLR4-/-, black bars) mice were cultured with media alone (M), ultra-pure lipopolysaccharide from *E*. *coli* (LPS, 1 μg/ml), extract of R03 (eR03, 100 μg/ml) or of ST1 (eST1, 100 μg/ml) for 18 h. Production of IL-12p70 (A), IL-10 (B), TNF-α (C) and IL-6 (D) in culture supernatants was measured by ELISA. Mean values ± SEM are shown. One representative out of three experiments yielding similar results is shown.

Incubation of wild-type BM-DC with eST1 led to significantly higher production of IL-12p70, TNF-α, IL-6 and IL-10 in comparison to eR03 (Fig 2A–2D). This cytokine production was TLR4 dependent and for the eST1 it was massively diminished in BM-DC derived from TLR4-/- mice. Concomitantly, eST1 is a potent inducer of TLR4-dependent maturation of BM-DC as shown by increased induction of CD40, CD80 and CD86 in comparison to unstimulated or eR03-incubated cells (Supporting Information S1 Fig).

### Contamination of sterile diet with endotoxin is associated with altered humoral response to Bet v 1 in germ-free mice

**Fig 3:**
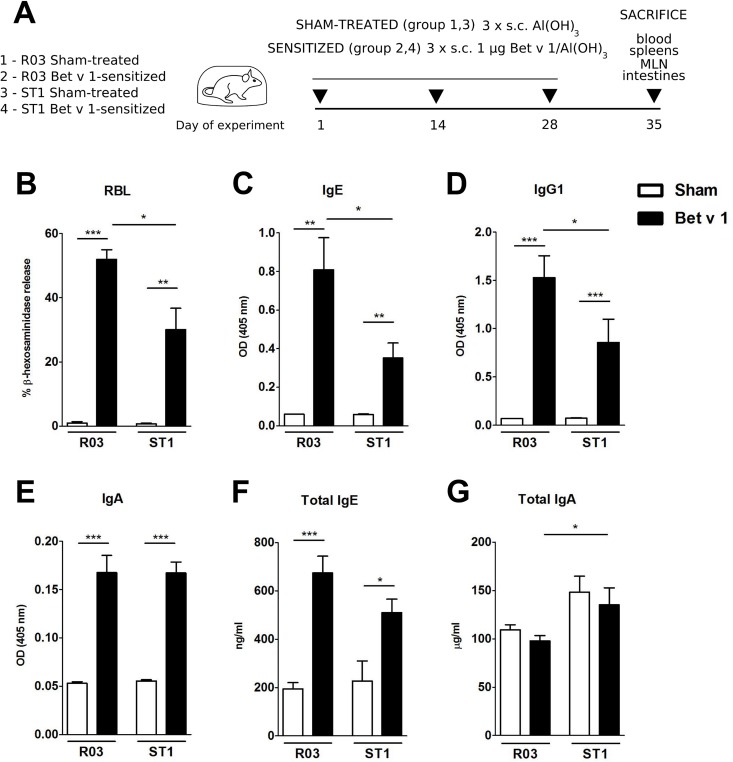
Systemic sensitization to Bet v 1 in mice bred on endotoxin-low (R03) and endotoxin-high (ST1) diet. (A) Experimental design: Mice were bred on the respective diet for at least two generations. Eight-week-old female germ-free mice fed with R03 or ST1 diet were sensitized by subcutaneous immunization (s.c.) three times with 1 μg of recombinant Bet v 1 in Alum (Bet v 1/Al(OH)_3_). Age-matched sham-treated mice were used as controls. At sacrifice, blood, spleens, and small intestines were collected for further analysis. (B) Functional IgE in serum was measured by Bet v 1-mediated β-hexosaminidase release from rat basophil leukemia cells. Bet v 1-specific IgE (C), IgG1 (D), IgA (E), total IgE (F) and total IgA (G) in sera were measured by ELISA. Data are plotted as mean values ± SEM. Pooled values of two independent experiments (n = 9–10 sensitized groups, n = 5–6 sham-treated groups) are shown. *P ≤ 0.05, **P ≤ 0.01, ***P ≤ 0.001.

**Fig 4:**
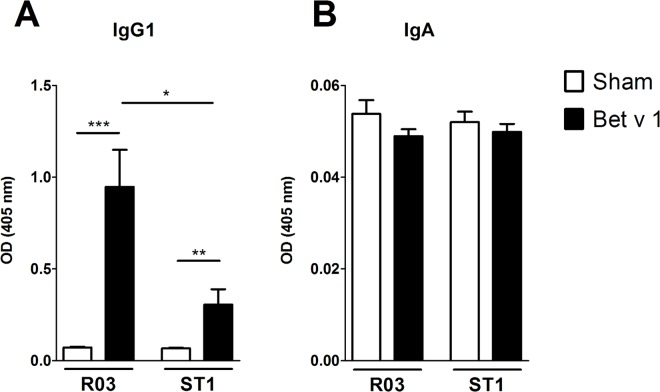
Levels of Bet v 1-specific IgG1 and IgA in gut lavage. Bet v 1-specific IgG1 (A) and IgA (B) in gut lavage were measured by ELISA. Data are plotted as mean values ± SEM. Pooled values of two independent experiments (n = 9–10 sensitized groups, n = 5–6 sham-treated groups) are shown. *P ≤ 0.05, **P ≤ 0.01, ***P ≤ 0.001.

Several studies have shown that mice raised in conventional conditions or mice mono-colonized with probiotic bacteria are less responsive to sensitization and display reduced levels of Th2-associated humoral responses compared to GF mice [17,22,24]. Here we tested whether food containing high levels of endotoxin can similarly influence the course of sensitization. We therefore investigated the production of specific and total antibodies after immunization with major birch pollen allergen Bet v 1 (Fig 3A). As expected, Bet v 1-immunization led to significant induction of specific antibodies in the serum in comparison to sham-treated controls (Fig 3B–3E). Interestingly, mice on ST1 diet exhibited significantly reduced Bet v 1-specific IgE-dependent ß-hexosaminidase release as well as lower production of specific IgE and IgG1 in the serum compared to sensitized GF animals on R03 diet (Fig 3B–3D). No significant differences were noted for Bet v 1-specific IgA in the serum between sensitized groups (Fig 3E). Also, there were no significant differences for Bet v 1-specific IgG2a in the serum between sensitized groups or sham-treated controls (data not shown). Levels of total IgE tend to be lower in the sensitized GF animals on ST1 diet in comparison to animals on R03 diet (Fig 3F). Feeding ST1 diet was associated with increased levels of total IgA in the serum and reached significant difference when sensitized groups on dissimilar diet were compared (Fig 3G). Decreased levels of Th2-associated antibodies in serum of sensitized animals fed on ST1 diet compared to R03-fed animals were accompanied with decreased levels of specific IgG1 in the gut lavage (Fig 4A). No significant differences were observed for specific IgA in the gut lavage among the groups (Fig 4B).

### Contamination of sterile diet with endotoxin is associated with altered antigen-specific and non-specific production of cytokines in spleen cell cultures

**Fig 5:**
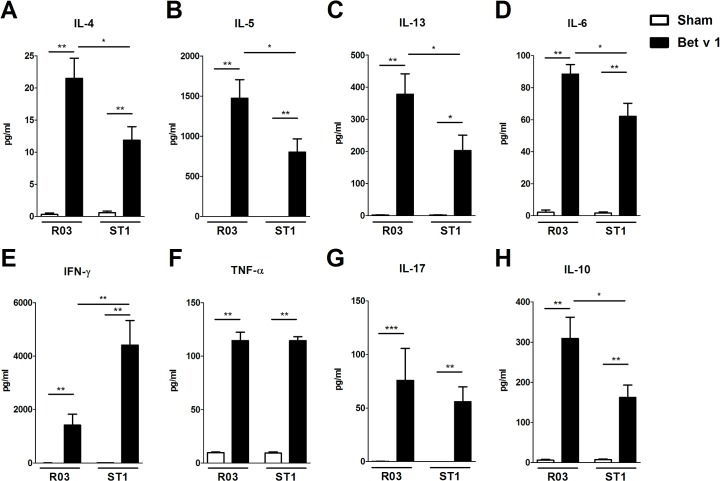
Influence of endotoxin-low (R03) and endotoxin-high (ST1) diet on Bet v 1-specific cytokine production in splenocytes. Germ-free mice fed endotoxin-low (R03) and endotoxin-high (ST1) diet were sensitized as indicated in Fig 2. Spleen cell cultures derived from these animals were incubated with 20 μg/ml of Bet v 1 for 60 h *in vitro*. Levels of IL-4 (A), IL-5 (B), IL-13 (C), IL-6 (D), IFN-γ (E), TNF-α (F), IL-17 (G), and IL-10 (H) in culture supernatants were measured by MILLIPLEX MAP Mouse Cytokine/Chemokine Panel. Cytokine levels are expressed after subtraction of base line levels of unstimulated splenocytes. Pooled values from two independent experiments (n = 9–10 sensitized groups, n = 5–6 sham-treated groups) are shown as mean values ± SEM. *P ≤ 0.05, **P ≤ 0.01, ***P ≤ 0.001.

**Fig 6:**
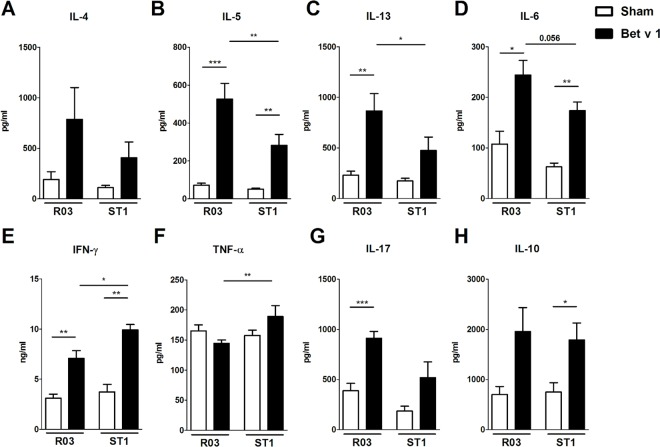
Influence of endotoxin-low (R03) and endotoxin-high (ST1) diet on mitogen-induced cytokine production in splenocytes. Germ-free mice fed endotoxin-low (R03) and endotoxin-high (ST1) diet were sensitized as indicated in Fig 2. Spleen cell cultures derived from these animals were incubated with 1.5 μg/ml of ConA for 60 h *in vitro*. Levels of IL-4 (A), IL-5 (B), IL-13 (C), IL-6 (D), IFN-γ (E), TNF-α (F), IL-17 (G) and IL-10 (H) in culture supernatants were measured by MILLIPLEX MAP Mouse Cytokine/Chemokine Panel. Cytokine levels are expressed after subtraction of base line levels of unstimulated splenocytes. Pooled values from two independent experiments (n = 9–10 sensitized groups, n = 5–6 sham-treated groups) are shown as mean values ± SEM. *P ≤ 0.05, **P ≤ 0.01, ***P ≤ 0.001.

To determine the role of contamination in diet on cellular responses in sensitized GF mice, we isolated spleens from sensitized and sham-treated mice fed R03 or ST1 diet. Single cell suspensions were cultured with/without Bet v 1 or with/without polyclonal mitogen ConA. Concerning the allergen-specific recall responses, the data clearly indicate that the presence of bacterial fragments in the diet was associated with reduced production of typical Th2-associated cytokines. In cell cultures derived from sensitized GF mice fed ST1 diet, the levels of Bet v 1-specific IL-4, IL-5, IL-13 and IL-10 in spleen cultures were significantly decreased in comparison to cultures derived from mice on R03 diet (Fig 5A–5C and 5H). On the other hand, the level of IFN-γ was increased in these mice. No differences were observed in levels of TNF-α and IL-17 (Fig 5F and 5G). Same trend has been observed for the Bet v 1-restimulated mesenteric lymph node cell cultures (data not shown). Regarding the non-specific cytokine responses induced by ConA in spleen cell cultures, similar picture was obtained as for Bet v 1-specific responses. ST1 diet was associated with reduced production of IL-5 and IL-13 (Fig 6B and 6C). Spleen cell cultures derived from sensitized ST1-fed animals produces significantly higher levels of IFN-γ and TNF-α in comparison to cells derived from R03-fed animals (Fig 6E and 6F). Production of ConA-induced IL-17 and IL-10 was comparable between R03 and ST1 sham or sensitized groups (Fig 6G and 6H).

## Discussion

Germ-free animals provide an attractive model for studying the host-microbiota interactions and they are extensively used in investigating the impact of gut microbiota on the maturation and function of the host immune system [20,35]. We and others have used GF animals to test the beneficial effects of single probiotic bacterial strains or their mixture on the development of several immune-mediated diseases [17,20,26,35–38]. Recently, it has been demonstrated that not only the presence of microbiota, such as bacteria, or macrobiota, such as helminth parasites in gastrointestinal tract, but also permanent exposure to self-antigens, ingested food antigens [27], viruses [39] or microbial residues from dead bacteria in sterile food or beddings, such as endotoxin, may influence the host immune responses. In this study, we tested whether signals derived from bacterial contamination in the sterile chow can influence the development of allergic sensitization to major birch pollen allergen Bet v 1 in germ-free mice. We could show that feeding on ST1 diet, which contains high levels of bacterial contamination such as endotoxin, reduced humoral and cellular responses to Bet v 1 when compared to animals fed on diet R03 with low content of endotoxin.

Several studies have shown that the immune responses in GF mice are skewed towards Th2 phenotype. For example, Hrncir *et al*. have detected increased ConA-induced production of IL-4 and decreased production of IFN-γ and IL-12 in spleen cell cultures of GF mice in comparison to conventionally reared animals [28]. Furthermore, Olszak *et al*. have shown increased pathology in model of asthma in GF mice compared to SPF controls [38]. Similarly, Hill at el. have shown that Th2 cell response are exaggerated in GF mice in comparison to animals colonized with commensal bacteria and depletion of bacterial communities by antibiotic treatment led to increased serum IgE and increased allergic inflammation [40].

We and others have previously demonstrated the potential of single probiotic bacteria or well defined bacterial mixtures to reverse the development of exacerbated allergic immune responses in germ-free mice [17,26,40]. These observations are in accordance with the concept of the hygiene hypothesis which proposes that exposure to microbes decreases susceptibility to atopic diseases. Although GF animals do not harbor any living microorganisms, they are still exposed to microbial residues from dead microorganisms in the sterile food or bedding [24]. We have measured the load of bacterial contamination in two different sterile diets ST1 and R03, which are commonly used in germ-free facilities. We could show that both the levels of bacterial DNA as well as level of endotoxin are markedly increased in ST1 in comparison to R03 diet. Using a PyroGene™Recombinant Factor C Assay, we determined that 1 mg of ST1 chow contains approximately 1200 EU of endotoxin. According to EC-5 US reference standard, 1 EU corresponds to 0.1 ng of endotoxin [41]. Thus, in average, a 20 g female mouse which consumes 2 g of ST1 chow/day, is exposed daily to approximately 0.24 mg of endotoxin. In previous study, Hrncir *et al*. have shown that LPS contamination in chow influenced the development and expansion of the host immune cells [28]. The question arising from this study was whether and to which extent the bacterial contamination impacts on the development of the course of allergic sensitization in a mouse model of type 1 allergy in germ-free conditions.

*In vitro*, we have shown that endotoxin from ST1 chow stimulates maturation of DC and induces production of pro-inflammatory cytokine IL-12p70 and regulatory IL-10. It has been previously shown that DC secreting IL-12 and/or IL-10 were able to efficiently inhibit the induction of allergic Th2 responses by inducing the differentiation of CD4+T cells towards a Th1 or Treg phenotype [42]. Along these lines decreased susceptibility for sensitization to allergens by systemic or local application of LPS was dependent on IL-12 production [13]. Furthermore, local administration of IL-12 resulted in decreased production of Th2 cytokines and the effect was associated with enhanced production of IFN-γ [43,44].

Epidemiological studies have shown that continuous exposure to endotoxin is associated with a lower prevalence of allergy in children [8]. For example, oral application of bacterial lysate containing Gram-negative *E*. *coli* led to reduced infantile atopic eczema [45]. In an animal model, oral application of bacterial lysate containing fecal *E*. *coli* led to reduced levels of allergen-specific IgE and IgG in serum in comparison to control animals [46]. Similarly, perinatal mucosal application of endotoxin prevented allergic sensitization and airway inflammation in mice [47]. Accordingly, we could show that exposure of mice to endotoxin in ST1 diet for at least two generations led to reduced levels of allergen-specific humoral and cellular responses in comparison to animals fed on R03 diet.

The feeding of high dose of endotoxin in ST1 diet was associated with decrease of Th2-associated cytokines and increase in production of allergen-specific IFN-γ in re-stimulated spleen cells. Our data are in agreement with study by Younger *et al*. that could show that LPS given orally to 25 g mice at range from 2.5 mg to 0.039 mg is effective to induce IFN-γ production [48]. Interestingly, in an experimental model of allergic airway inflammation, inhalation of LPS together with an allergen has been linked to the development of allergy in both protecting and facilitating role, where low level of LPS was indispensable for Th2 priming, while high dose LPS reduced inflammatory responses in a mouse model of allergic airway inflammation [49]. The question whether low level of bacterial fragments presented in the diet R03 could play a role in induction of Th2 sensitization remains to be evaluated.

Further, we have shown that higher bacterial contamination in the diet leads to decreased level of allergen-specific IgE and IgG1 antibodies in sera accompanied by decreased levels of specific IgG1 in small intestine, suggesting lower level of sensitization in these mice. This data are in agreement with our previous study where colonization of GF mice with a mixture of 3 *Lactobacillus* strains prevented the development of allergic sensitization associated with reduced levels of IgE and IgG1 [26]. It has been shown that immature B cells preferentially switch to IgE [50] and signals derived from the intestinal microbial colonization have been found to influence the immunoglobulin repertoires in the gut lamina propria [51]. Previous studies investigating the impact of microbiota on sensitization in germ-free animals produced contrasting data. According to Hazebrouck *et al*. and Rodriguez et al., sensitization in GF mice on R03 diet led to increased levels of allergen-specific humoral and cellular responses in comparison to conventional mice [22,24]. On the contrary, study by Repa *et al*. showed that the levels of specific humoral immune response are independent on microbial colonization in mice on ST1 diet [52]. Certainly, the discrepancy between these studies might result from the different sensitization protocol and/or the different allergen used. However, in the light of our recent data; it is tempting to speculate that this difference might be due to the endotoxin contamination in the diet. Therefore, in agreement with Reliene and Schiestl [53], we suggest that original scientific articles should supply information of the type of diet used.

Taken together our findings expand on the hygiene hypothesis. We suggest that not only intestinal microbiota or parasites influence the development of allergic responses in experimental settings, but also bacterial fragments in the sterile diet may have a profound effect on level of sensitization under germ-free conditions. Importantly, observations from our study may be relevant to germ-free or gnotobiotic experimental models investigating the role of microbiota in several other models of immune-deviated inflammatory diseases.

## Supporting Information

S1 Fig Flow cytometry analyses of maturation of bone marrow-derived DC. Mouse bone marrow-derived DC (BM-DC) were prepared and stimulated as described in the manuscript. BM-DC were labelled with monoclonal antibodies for CD11c (FITC), MHC II (APC), CD40, CD80 or CD86 (PE) (eBioscience, USA). Appropriate isotype antibodies were used as controls to determine non-specific binding. Cells were analyzed using FACSCalibur flow cytometer (Becton-Dickinson, USA) and obtained data were analyzed with FlowJo 7.6.2 software (TreeStar, USA).
(PNG)

S1 Table Composition of the feed mixture ST1. (DOCX)

## Abbreviations

GF: germ-free
TLR: Toll like receptor
ConA: Concanavalin A
DC: dendritic cells
Th: T helper
GALT: gastrointestinal associated lymphoid tissue
SPF: specific pathogen free
BM-DC: bone marrow-derived dendritic cells
HEK293 cells: human embryonic kidney cells
Pam3Cys: Pam3CSK4

## References

[1] Wills-Karp M, Santeliz J, Karp CL. The germless theory of allergic disease: Revisiting the Hygiene Hypothesis. Nat Reviews Immunol. 2001;1: 69–75. 10.1038/35095579 11905816

[2] Abrahamsson TR, Jakobsson HE, Andersson AF, Björksten B, Engstrand L, Jenmalm MC, et al. Low gut microbiota diversity in early infancy precedes asthma at school age. Clin Exp Allergy. 2014;44: 842–850. 10.1111/cea.12253 24330256

[3] Kalliomäki M, Kirjavainen P, Eerola E, Kero P, Salminen S, Isolauri E. Distinct patterns of neonatal gut microflora in infants in whom atopy was and was not developing. J Allergy Clin Immunol. 2001;107: 129–134. 10.1067/mai.2001.111237 11150002

[4] Stiemsma LT, Reynolds LA, Turvey SE, Finlay BB. The hygiene hypothesis: Current perspectives and future therapies. ImmunoTargets Ther. 2015;4: 143–157. 10.2147/ITT.S61528 27471720 PMC4918254

[5] Braun-Fahrländer C, Riedler J, Herz U, Eder W, Waser M, Grize L, et al. ENVIRONMENTAL EXPOSURE TO ENDOTOXIN AND ITS RELATION TO ASTHMA IN SCHOOL-AGE CHILDREN. N Engl J Med. 2002;347: 869–77. 10.1056/NEJMoa020057 12239255

[6] Gehring U, Bischof W, Fahlbusch B, Wichmann HE, Heinrich J. House dust endotoxin and allergic sensitization in children. Am J Respir Crit Care Med. 2002;166: 939–944. 10.1164/rccm.200203-256OC 12359650

[7] Garn H, Renz H. Epidemiological and immunological evidence for the hygiene hypothesis. Immunobiology. 2007;212: 441–452. 10.1016/j.imbio.2007.03.006 17544829

[8] Simpson A, Martinez FD. The role of lipopolysaccharide in the development of atopy in humans: review. Clin Exp Allergy. 2010;40: 209–223. 10.1111/j.1365-2222.2009.03391.x 19968655

[9] Riedler J, Braun-Fahrländer C, Eder W, Schreuer M, Waser M, Maisch S, et al. Exposure to farming in early life and development of asthma and\rallergy: a cross-sectional survey. Lancet. 2001;358: 1129–1133. 10.1016/S0140-6736(01)06252-3 11597666

[10] Sordillo JE, Hoffman EB, Celedón JC, Litonjua AA, Milton DK, Gold DR. Multiple microbial exposures in the home may protect against asthma or allergy in childhood. Clin Exp Allergy. 2010;40: 902–910. 10.1111/j.1365-2222.2010.03509.x 20412140 PMC3730840

[11] Cunha FQ, Lefort J, Vargaftig BB, Russo M, Rodríguez D, Keller AC, et al Bacterial Lipopolysaccharide Signaling Through Toll-Like Receptor 4 Suppresses Asthma-Like Responses Via Nitric Oxide Synthase 2 Activity 1. J immunol. 2003;171: 1001–108. 12847273 10.4049/jimmunol.171.2.1001

[12] Blümer N, Herz U, Wegmann M, Renz H. Prenatal lipopolysaccharide-exposure prevents allergic sensitization and airway inflammation, but not airway responsiveness in a murine model of experimental asthma. Clin Exp Allergy. 2005;35: 397–402. 10.1111/j.1365-2222.2005.02184.x 15784121

[13] Gerhold K, Blumchen K, Bock A, Seib C, Stock P, Kallinich T, et al. Endotoxins prevent murine IgE production, TH2 immune responses, and development of airway eosinophilia but not airway hyperreactivity. J Allergy ClinImmunol. 2002;110: 110–116. 10.1067/mai.2002.125831 12110829

[14] Debarry J, Hanuszkiewicz A, Stein K, Holst O, Heine H. The allergy-protective properties of Acinetobacter lwoffii F78 are imparted by its lipopolysaccharide. Allergy Eur J Allergy Clin Immunol. 2010;65: 690–697. 10.1111/j.1398-9995.2009.02253.x 19909295

[15] Debarry J, Garn H, Hanuszkiewicz A, Dickgreber N, Blumer N, von Mutius E, et al Acinetobacter lwoffii and Lactococcus lactis strains isolated from farm cowsheds possess strong allergy-protective properties. J Allergy Clin Immunol. 2007;119: 1514–1521. 10.1016/j.jaci.2007.03.023 17481709

[16] Park BS, Song DH, Kim HM, Choi B-S, Lee H, Lee J-O. The structural basis of lipopolysaccharide recognition by the TLR4-MD-2 complex. Nature. Nature Publishing Group; 2009;458: 1191–5. 10.1038/nature07830 19252480

[17] Schwarzer M, Srutkova D, Schabussova I, Hudcovic T, Akgün J, Wiedermann U, et al Neonatal colonization of germ-free mice with Bifidobacterium longum prevents allergic sensitization to major birch pollen allergen Bet v 1. Vaccine. Elsevier Ltd; 2013;31: 5405–12. 10.1016/j.vaccine.2013.09.014 24055352

[18] Trujillo C, Erb KJ. Inhibition of allergic disorders by infection with bacteria or the exposure to bacterial products. Int J Med Microbiol. 2003;293: 123–131. 10.1078/1438-4221-00257 12868649

[19] Bortolatto J, Borducchi E, Rodriguez D, Keller AC, Faquim-Mauro E, Bortoluci KR, et al. Toll-like receptor 4 agonists adsorbed to aluminium hydroxide adjuvant attenuate ovalbumin-specific allergic airway disease: Role of MyD88 adaptor molecule and interleukin-12/interferon-γ axis. Clin Exp Allergy. 2008;38: 1668–1679. 10.1111/j.1365-2222.2008.03036.x 18631348

[20] Tlaskalová-Hogenová H, Štěpánková R, Kozáková H, Hudcovic T, Vannucci L, Tučková L, et al. The role of gut microbiota (commensal bacteria) and the mucosal barrier in the pathogenesis of inflammatory and autoimmune diseases and cancer: contribution of germ-free and gnotobiotic animal models of human diseases. Cell Mol Immunol. 2011;8: 110–120. 10.1038/cmi.2010.67 21278760 PMC4003137

[21] Lane DJ. 16S/23S rRNA sequencing In: Stackebrandt E., and Goodfellow M, editor. Nucleic acid techniques in bacterial systematics. John Wiley and Sons, New York, NY; 1991 pp. 115–175.

[22] Rodriguez B, Prioult G, Bibiloni R, Nicolis I, Mercenier A, Butel M-J, et al. Germ-free status and altered caecal subdominant microbiota are associated with a high susceptibility to cow’s milk allergy in mice. FEMS Microbiol Ecol. 2011;76: 133–144. 10.1111/j.1574-6941.2010.01035.x 21223329

[23] Stefka AT, Feehley T, Tripathi P, Qiu J, McCoy K, Mazmanian SK, et al. Commensal bacteria protect against food allergen sensitization. Proc Natl Acad Sci U S A. 2014;111: 13145–50. 10.1073/pnas.1412008111 25157157 PMC4246970

[24] Hazebrouck S, Przybylski-Nicaise L, Ah-Leung S, Adel-Patient K, Corthier G, Wal JM, et al Allergic sensitization to bovine beta-Lactoglobulin: Comparison between germ-free and conventional BALB/c mice. Int Arch Allergy Immunol. 2008;148: 65–72. 10.1159/000151507 18716405

[25] Herbst T, Sichelstiel A, Schär C, Yadava K, Bürki K, Cahenzli J, et al. Dysregulation of Allergic Airway Inflammation in the Absence of Microbial Colonization. Am J Respir Crit Care Med. 2011;184: 198–205. 10.1164/rccm.201010-1574OC 21471101

[26] Kozakova H, Schwarzer M, Tuckova L, Srutkova D, Czarnowska E, Rosiak I, et al. Colonization of germ-free mice with a mixture of three lactobacillus strains enhances the integrity of gut mucosa and ameliorates allergic sensitization. Cell Mol Immunol. Nature Publishing Group; 2016;13: 251–262. 10.1038/cmi.2015.09 25942514 PMC4786630

[27] Kim KS, Hong S-W, Han D, Yi J, Jung J, Yang B-G, et al. Dietary antigens limit mucosal immunity by inducing regulatory T cells in the small intestine. Science (80-). 2016;351: 858–863. 10.1126/science.aac5560 26822607

[28] Hrncir T, Stepankova R, Kozakova H, Hudcovic T, Tlaskalova-Hogenova H. Gut microbiota and lipopolysaccharide content of the diet influence development of regulatory T cells: studies in germ-free mice. BMC Immunol. 2008;9: 65 10.1186/1471-2172-9-65 18990206 PMC2588440

[29] Srutkova D, Spanova A, Spano M, Drab V, Schwarzer M, Kozakova H, et al Efficiency of PCR-based methods in discriminating Bifidobacterium longum ssp. longum and Bifidobacterium longum ssp. infantis strains of human origin. J Microbiol Methods. 2011;87: 10–16. 10.1016/j.mimet.2011.06.014 21756944

[30] Schwarzer M, Makki K, Storelli G, Machuca-Gayet I, Srutkova D, Hermanova P, et al. Lactobacillus plantarum strain maintains growth of infant mice during chronic undernutrition. Science (80-). 2016;351: 854–857. 10.1126/science.aad8588 26912894

[31] Takakuwa T, Knopf HP, Sing A, Carsetti R, Galanos C, Freudenberg MA. Induction of CD14 expression in Lps(n), Lps(d) and tumor necrosis factor receptor-deficient mice. Eur J Immunol. 1996;26: 2686–2692. 10.1002/eji.1830261121 8921956

[32] Schwarzer M, Repa A, Daniel C, Schabussova I, Hrncir T, Pot B, et al. Neonatal colonization of mice with Lactobacillus plantarum producing the aeroallergen Bet v 1 biases towards Th1 and T-regulatory responses upon systemic sensitization. Allergy Eur J Allergy Clin Immunol. 2011;66: 368–375. 10.1111/j.1398-9995.2010.02488.x 20880132

[33] Schabussova I, Hufnagl K, Wild C, Nutten S, Zuercher AW, Mercenier A, et al. Distinctive anti-allergy properties of two probiotic bacterial strains in a mouse model of allergic poly-sensitization. Vaccine. Elsevier Ltd; 2011;29: 1981–1990. 10.1016/j.vaccine.2010.12.101 21216308

[34] Schabussova I, Hufnagl K, Tang ML, Hoflehner E, Wagner A, Loupal G, et al Perinatal maternal administration of Lactobacillus paracasei NCC 2461 prevents allergic inflammation in a mouse model of birch pollen allergy. PLoS One. 2012;7: e40271 10.1371/journal.pone.0040271 22792257 PMC3391241

[35] Gordon HA, Pesti L. The gnotobiotic animal as a tool in the study of host microbial relationships. Bact Rev. 1971;35: 390–429. 4945725 10.1128/br.35.4.390-429.1971 PMC378408

[36] Du Z, Hudcovic T, Mrazek J, Kozakova H, Srutkova D, Schwarzer M, et al Development of gut inflammation in mice colonized with mucosa-associated bacteria from patients with ulcerative colitis. Gut Pathog. BioMed Central; 2015;7: 32 10.1186/s13099-015-0080-2 26697117 PMC4687314

[37] Cinova J, de Palma G, Stepankova R, Kofronova O, Kverka M, Sanz Y, et al Role of intestinal bacteria in gliadin-induced changes in intestinal mucosa: Study in germ-free rats. PLoS One. 2011;6. 10.1371/journal.pone.0016169 PMC3020961 21249146

[38] Olszak T, An D, Zeissig S, Vera MP, Richter J, Franke A, et al. Microbial exposure during early life has persistent effects on natural killer T cell function. Science. 2012;336: 489–493. 10.1126/science.1219328 22442383 PMC3437652

[39] Kernbauer E, Ding Y, Cadwell K. An enteric virus can replace the beneficial function of commensal bacteria. Nature. Nature Publishing Group; 2014;516: 94–98. 10.1038/nature13960 25409145 PMC4257755

[40] Hill DA, Siracusa MC, Abt MC, Kim BS, Kobuley D, Kubo M, et al. Commensal bacteria–derived signals regulate basophil hematopoiesis and allergic inflammation. Nat Med. Nature Publishing Group; 2012;18: 538–546. 10.1038/nm.2657 22447074 PMC3321082

[41] Zhu Z, Oh SY, Zheng T, Kim YK. Immunomodulating effects of endotoxin in mouse models of allergic asthma. Clin Exp Allergy. 2010;40: 536–546. 10.1111/j.1365-2222.2010.03477.x 20447074

[42] Bilenki L, Gao X, Wang S, Yang J, Fan Y, Han X, et al. Dendritic cells from mycobacteria-infected mice inhibits established allergic airway inflammatory responses to ragweed via IL-10- and IL-12-secreting mechanisms. J Immunol. 2010;184: 7288–96. 10.4049/jimmunol.0902829 20483754

[43] Préfontaine D, Banville-Langelier AA, Fiset PO, Guay J, An J, Mazer M, et al. Children with atopic histories exhibit impaired lipopolysaccharide-induced Toll-like receptor-4 signalling in peripheral monocytes. Clin Exp Allergy. 2010;40: 1648–1657. 10.1111/j.1365-2222.2010.03570.x 20636402

[44] Gavett BSH, Hearn DO, Li X, Huang S, Finkelman FD. Interleukin 12 Inhibits Antigen-induced Airway Hyperresponsiveness, Inflammation, and Th2 Cytokine Expression in Mice. 1995;182: 1527–1536. 7595222 10.1084/jem.182.5.1527 PMC2192202

[45] Lau S, Gerhold K, Zimmermann K, Ockeloen CW, Rossberg S, Wagner P, et al. Oral application of bacterial lysate in infancy decreases the risk of atopic dermatitis in children with 1 atopic parent in a randomized, placebo-controlled trial. J Allergy Clin Immunol. Elsevier Ltd; 2012;129: 1040–1047. 10.1016/j.jaci.2012.02.005 22464674

[46] Ahrens B, Quarcoo D, Buhner S, Matricardi PM, Hamelmann E. Oral administration of bacterial lysates attenuates experimental food allergy. Int Arch Allergy Immunol. 2011;156: 196–204. 10.1159/000322352 21597300

[47] Gerhold K, Avagyan A, Seib C, Frei R, Steinle J, Ahrens B, et al. Prenatal initiation of endotoxin airway exposure prevents subsequent allergen-induced sensitization and airway inflammation in mice. J Allergy Clin Immunol. 2006;118: 666–73. 10.1016/j.jaci.2006.05.022 16950286

[48] Youngner JS. Bacterial lipopolysaccharide: oral route for interferon production in mice. Infect Immun. 1972;6: 646–647. 4628903 10.1128/iai.6.4.646-647.1972 PMC422587

[49] Eisenbarth SC, Piggott DA, Huleatt JW, Visitin I, Herrick CA, Bottomly K. Lipopolysaccharide-enhanced, Toll-like receptor 4-dependent T helper cell type 2 responses to inhaled antigen. J. Exp. Med. 2002; 196:12: 1645–1651. 10.1084/jem.20021340 12486107 PMC2196061

[50] Wesemann DR, Magee JM, Boboila C, Calado DP, Gallagher MP, Portuguese AJ, et al Immature B cells preferentially switch to IgE with increased direct S to S recombination. J Exp Med. 2011;208: 2733–2746. 10.1084/jem.20111155 22143888 PMC3244039

[51] Wesemann DR, Portuguese AJ, Meyers RM, Gallagher MP, Cluff-Jones K, Magee JM, et al. Microbial colonization influences early B-lineage development in the gut lamina propria. Nature. Nature Publishing Group; 2013;501: 112–5. 10.1038/nature12496 23965619 PMC3807868

[52] Repa A, Kozakova H, Hudcovic T, Stepankova R, Hrncir T, Tlaskalova-Hogenova H, et al. Susceptibility to nasal and oral tolerance induction to the major birch pollen allergen Bet v 1 is not dependent on the presence of the microflora. Immunol Lett. 2008;117: 50–56. 10.1016/j.imlet.2007.11.025 18241932

[53] Reliene R. and Schiestl RH. Differences in animal housing facilities and diet may affect study outcomes–a plea for inclusion of such information in publication. DNA Repair. 2006; 651–653. 10.1016/j.dnarep.2006.02.001 16581314

