# Supplementary figures and images for: Diet Matters: Endotoxin in the Diet Impacts the Level of Allergic Sensitization in Germ-Free Mice
Source: PLoS One. 2017 Jan 4;12(1):e0167786. doi: 10.1371/journal.pone.0167786 (PMC5215724; doi:10.1371/journal.pone.0167786)

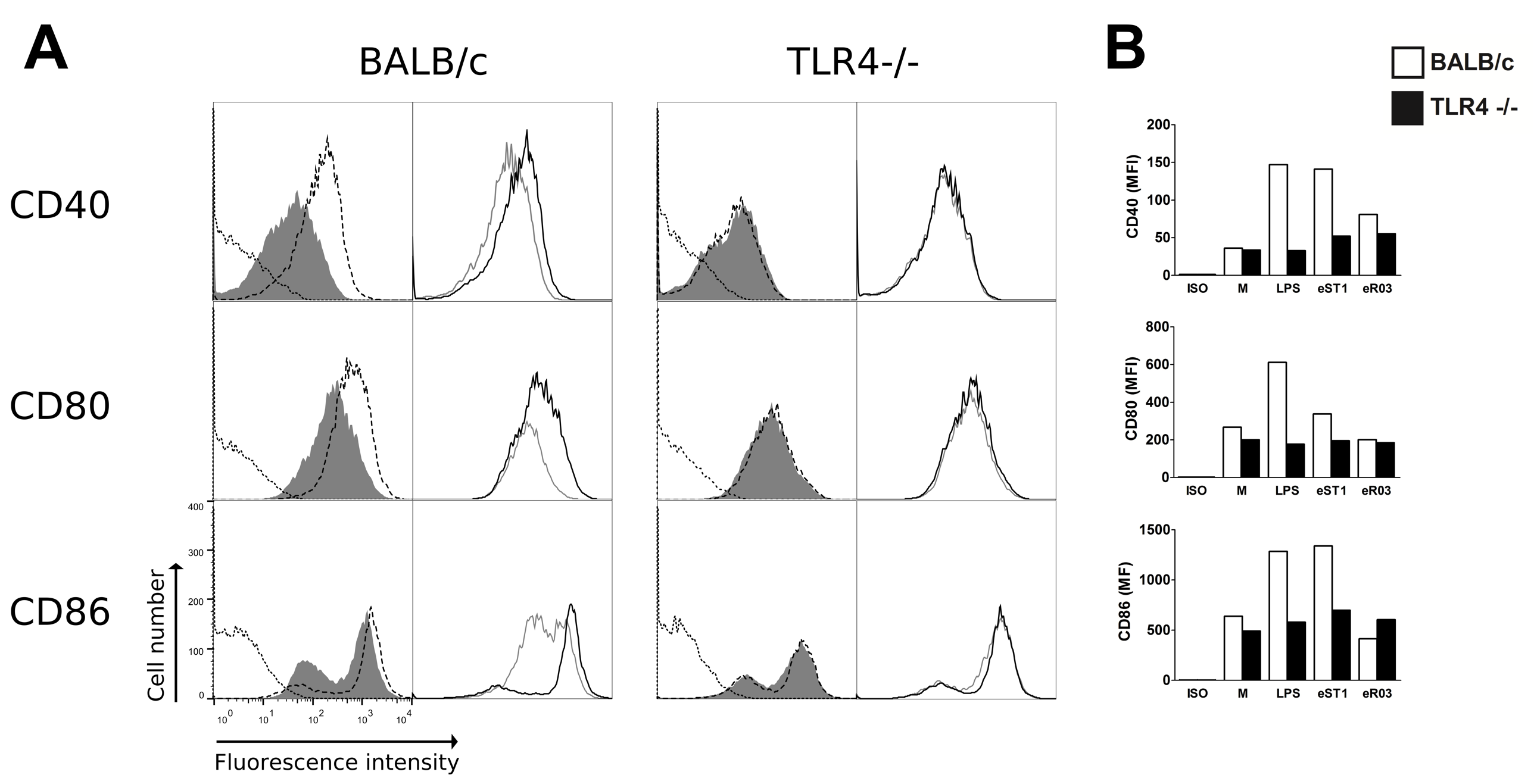

Supplement: S1 Fig — Mouse bone marrow-derived DC (BM-DC) were prepared and stimulated as described in the manuscript. BM-DC were labelled with monoclonal antibodies for CD11c (FITC), MHC II (APC), CD40, CD80 or CD86 (PE) (eBioscience, USA). Appropriate isotype antibodies were used as controls to determine non-specific binding. Cells were analyzed using FACSCalibur flow cytometer (Becton-Dickinson, USA) and obtained data were analyzed with FlowJo 7.6.2 software (TreeStar, USA). (PNG) [file pone.0167786.s001.png]
