# Supplementary material for: Diet Matters: Endotoxin in the Diet Impacts the Level of Allergic Sensitization in Germ-Free Mice
Source: PLoS One. 2017 Jan 4;12(1):e0167786. doi: 10.1371/journal.pone.0167786 (PMC5215724; doi:10.1371/journal.pone.0167786)
Supplement: S1 Table — (DOCX) [file pone.0167786.s002.docx]

Supplementary Table S1:

**Composition of the feed mixture ST1**

Ingredients: Ground wheat, ground oats, ground corn, wheat meal, dehydrated alfalfa meal, soybean meal, fish meal, feeding calcite, dicalcium phospate, feeding salt, methionin, vitamins.

Average nutrient composition in 1kg (from www.velaz.cz):

Moisture 12,50%

Protein 24,00%

Fat 3,40%

Fiber 4,40%

Dry matter 6,80%

Lysin 14,00 g

Methionin 4,80 g

Calcium 11,00 g

Phosphorus 7,20 g

Natrium 1,80 g

Copper 20,00 mg/kg

Selenium 0,38 mg/kg

Vitamin A 28000,00 IU/kg

Vitamin D3 2200,00 IU/kg

Vitamin E 100,00 mg/kg
